# Picoeukaryotic Diversity And Activity in the Northwestern Pacific Ocean Based on rDNA and rRNA High-Throughput Sequencing

**DOI:** 10.3389/fmicb.2018.03259

**Published:** 2019-01-09

**Authors:** Feipeng Wang, Yuyuan Xie, Wenxue Wu, Ping Sun, Lei Wang, Bangqin Huang

**Affiliations:** ^1^Fujian Provincial Key Laboratory of Coastal Ecology and Environmental Studies, State Key Laboratory of Marine Environmental Science, Xiamen University, Xiamen, China; ^2^School of Marine Sciences, Sun Yat-sen University, Zhuhai, China; ^3^Third Institute of Oceanography, State Oceanic Administration, Xiamen, China

**Keywords:** picoeukaryotic diversity, Illumina sequencing, 18S rRNA gene and cDNA sequencing, RNA/DNA comparison, metabolic activity, northwestern Pacific Ocean

## Abstract

Picoeukaryotes play an important role in the biogenic element cycle and energy flow in oligotrophic ecosystems. However, their biodiversity and activity are poorly studied in open ocean systems, such as the northwestern Pacific Ocean, which is characterized by a complex hydrological setting. Here, we investigated the diversity and activity of picoeukaryotes in the northwestern Pacific Ocean using high-throughput sequencing targeting the V9 region of 18S rDNA and rRNA. Our results showed that the DNA picoeukaryotic communities were mainly represented by Mamiellophyceae, MAST, MALV-II, Spirotrichea, Prymnesiophyceae, and MALV-I (69.33% of the total DNA reads), and the RNA communities were dominated by Spirotrichea, Mamiellophyceae, MAST, Pelagophyceae, and MALV-II (67.46% of the total RNA reads). The number of operational taxonomic units (OTUs) was significantly affected by temperature and salinity, and was decreased with the increasing nutrient concentration both in the DNA and RNA surveys. Significant differences were observed in the community composition between DNA-based and RNA-based molecular approaches, and these differences were mainly attributed to Mamiellophyceae, Spirotrichea, and Pelagophyceae. The RNA: DNA ratio was used as a proxy for relative metabolic activity of the individual OTUs. We found that the relative metabolic activities of Mamiellophyceae, Spirotrichea, and Pelagophyceae species in the northwestern Pacific Ocean were highly affected by the nutrient concentration, i.e., the NO_3_ + NO_2_ and SiO_3_ concentration. Overall, our study shed light on picoeukaryotic diversity and distribution in the northwestern Pacific Ocean and revealed the correlation between the diversity, relative metabolic activities of marine picoeukaryotes, and the environmental factors.

## Introduction

Picoeukaryotes (cell sizes at 0.2–3 μm) are key components of marine ecosystems and microbial food webs (Pomeroy et al., 2007; Vaulot et al., 2008; Caron et al., 2012). Picoeukaryotes are widely distributed and consist of multiple metabolic types, including phototrophs, heterotrophs, and mixotrophs (Zubkov and Tarran, 2008; de Vargas et al., 2015). Photosynthetic picoeukaryotes (PPEs) are vital contributors of marine plankton biomass and primary production in coastal and oceanic environments (Worden et al., 2004; Grob et al., 2007; Jardillier et al., 2010). Heterotrophic picoeukaryotes within Stramenopiles and Alveolates can feed on bacteria and therefore play an important role in nutrient recycling (from prokaryotic fractions to higher trophic levels, Sherr and Sherr, 1994; Massana et al., 2009), as well as bacterial community composition and abundance (Jardillier et al., 2005). Recent studies have expanded our knowledge of the ecological roles of mixotrophic picoeukaryotes, showing that they are important consumers of prokaryotes and have the potential to dominate the primary production and bacterivory in marine ecosystems (Hartmann et al., 2012; Sanders and Gast, 2012; Unrein et al., 2014).

Using the sequencing of environmental DNA (eDNA) based on the 18S rRNA gene, previous studies reveled an astonishing picoeukaryotic diversity in aquatic environments (Díez et al., 2001; Moon-van der Staay et al., 2001; Berney et al., 2004; Massana et al., 2004; Richards et al., 2005; Not et al., 2007). With the development of sequencing technologies, high throughput sequencing (HTS) highlights the phylogenetic diversity of protist communities with high-resolution exploration (Zinger et al., 2011; Massana et al., 2015; Hu et al., 2016; Xu et al., 2017; Bellaaj Zouari et al., 2018). rRNA gene sequencing (hereafter, referred to as DNA) has been commonly used for the investigation of microbial diversity in environmental samples (Moon-van der Staay et al., 2001; Shi et al., 2009). However, microbial community structures inferred from DNA sequencing can be distorted by extracellular free DNA (Karl and Bailiff, 1989; Massana et al., 2015), dormant cells or fragments of dead materials (Stoeck et al., 2007; Not et al., 2009). In contrast, rRNA (hereafter, referred to as RNA) is much more unstable in extracellular conditions, and sequence information derived from RNA can imply ribosomal activity and the potential of protein synthesis (Not et al., 2009; Blazewicz et al., 2013; Egge et al., 2015). RNA molecular approach is nowadays generally accepted to identify the active protist community (Massana et al., 2015), although several critical limitations should be carefully considered (Blazewicz et al., 2013). Thus, RNA sequencing can act as a supplement to DNA sequencing to achieve a better understanding of picoeukaryotic communities in the natural environment (Not et al., 2009; Terrado et al., 2011). The RNA: DNA ratio (based on relative abundances) have been used as a proxy for the relative metabolic activity in protist (Massana et al., 2015; Hu et al., 2016; Xu et al., 2017), while this strategy needed to be applied with caution (Blazewicz et al., 2013; Hu et al., 2016). Significant changes of the RNA: DNA ratio may indicate ecological season events. A recent study have found that the changes of RNA: DNA ratio of *Phaeocystis globose*, collected at a coastal station of the eastern English Channel, unveiled well to the *Phaeocystis* bloom dynamic (Rachik et al., 2018).

The northwestern Pacific Ocean is a complex system of multiple ocean currents, including the Kuroshio Current (KC), Kuroshio Extension Current (KEC), and Oyashio Current (OC). The KC and its extension (KEC) are characterized by warm and saline waters (salinity above 34.2) from the southern subtropical area, and they flow in a northeastern direction. The OC transports cold waters of lower salinities (below 33.5; Qiu, 2001) from the north subarctic area and flows to the southwest. The Kuroshio-Oyashio mixing region is the location where the warm subtropical KEC waters encounter the cold subarctic OC waters, roughly ranging from 36 to 44°N (Chen, 2008). Hydrographical characteristics in this area exhibit complicated frontal structures, tongues, and eddies (Yasuda, 2003). Several previous studies have revealed the response of picoplankton diversity to changing environmental factors, e.g., temperature, nutrient content, and oxygen concentration (Doney et al., 2012; Grossmann et al., 2016). However, only a few studies have focused on picoeukaryotic community structures in the northwestern Pacific Ocean, and the correlation between varied environmental factors and picoeukaryotic diversity in this area has rarely been examined (Liu et al., 2002; Choi et al., 2016). Nishibe et al. (2015, 2017) revealed that phytoplankton communities across the KEC during spring mainly comprised chlorophytes, cryptophytes, and prasinophytes (based on pigment analysis). The small phytoplankton groups (< 10 μm) were the main contributors for the phytoplankton biomass and primary production (Nishibe et al., 2015). To the best of our knowledge, this was the first study of the investigation of picoeukaryotic diversity and activity in the northwestern Pacific Ocean based on HTS (DNA and RNA). The aim of this study was to: (1) reveal picoeukaryotic assemblages in the northwestern Pacific Ocean based on rDNA and rRNA sequencing, and (2) examine the extent to which environmental factors influenced picoeukaryotic diversity and relative metabolic activity.

## Materials and Methods

### Cruise, Sampling, and Measurement of Environmental Parameters

**Figure 1:**
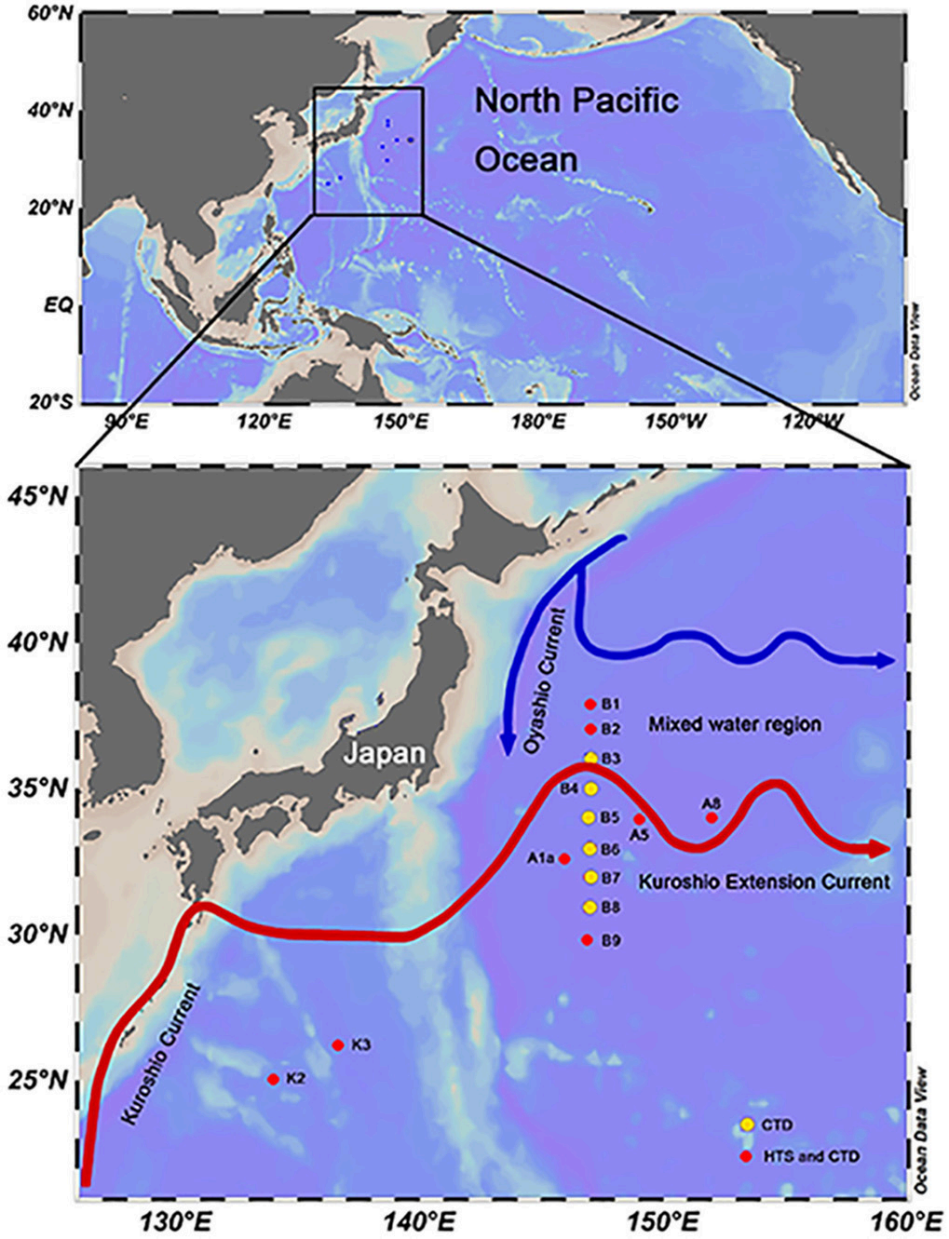
Sampling sites in the northwestern Pacific Ocean during the spring cruise conducted in 2015. Flow paths of Kuroshio and its Extension Current and the Oyashio Current were modified according to the study of Qiu (2001). The red dots on the map indicate the high-throughput sequencing stations. The yellow dots on the map indicate stations within the B transect for which only physical and chemical parameters were recorded by CTD. The map was produced using the Ocean Data View 4 software.

HTS samples were taken from 8 stations in the northwestern Pacific Ocean, from 134 to 152°E and 25 to 38°N from March 29th to May 4th, 2015 (Figure 1). For each station, 20–30 L seawater was collected from both the surface (Sur, 5 m) and deep chlorophyll maximum (DCM) layers using Niskin bottles mounted on a CTD (conductivity, temperature, and depth) rosette (Sea-Bird Electronics, USA). The seawater was sequentially filtered using a peristaltic pump through 3 and 0.2 μm pore size polycarbonate membranes (142 mm diameter, Millipore, USA) at a rate of 0.5–1 L/min. The filters (samples collected between 0.2 and 3 μm) were transferred into a 5 mL cryo tube containing 4.6 mL of RNA*later* (Thermo Fisher, Lithuania). The tubes were immediately frozen in liquid nitrogen on board. In this study, HTS samples were named by the combination of sampling station and depth, e.g., K2Sur indicates that the sample was collected at the surface layer of Stn. K2. Environmental parameters (i.e., temperature, salinity, and depth) were recorded with SBE-911 CTD (Sea-Bird Electronics, USA) in each sampling site including the B transect (Figure 1). The samples for pigment analyses were also collected in the Sur and DCM layers of the 8 HTS stations. For the pigment measurements, 4–6 L of seawater without pre-filtration were directly filtered onto 25 mm GF/F glass microfiber filters (Whatman, USA) under 200 mm Hg pressure. These filters were then protected from the light and immediately stored in liquid nitrogen on board, kept at −80°C in the laboratory until analysis. Photosynthetic pigment concentrations were measured by high-performance liquid chromatography (HPLC) (Zapata et al., 2000). The chlorophyll *a* (Chl *a*) concentrations were derived from the pigment analysis. The CHEMical TAXonomy (CHEMTAX, Mackey et al., 1996) was applied to acquire the relative contributions of different phytoplankton groups to the total Chl *a*. Samples for flow cytometry analysis were also collected in the HTS sampling sites. 1.8 ml seawater was transferred into a 2 ml cryo tube, and then fixed with paraformaldehyde (1% final concentrations). The tubes were incubated at room temperature for 15 min and then frozen in liquid nitrogen on board. Estimation of the cell abundance of picoeukaryotes was performed by a Becton Dickinson FACSCalibur flow cytometer.

### DNA and RNA Co-extraction, PCR Amplification, and Sequencing

The total DNA and RNA were extracted simultaneously from each sample using the AllPrep DNA/RNA Mini Kit (Qiagen, #80204, Germany). For the RNA extraction, gDNA wipeout buffer (Qiagen, #205311, Germany) was used to remove the genomic DNA. The purified RNA was reverse transcribed into cDNA using the QuantiTect Reverse Transcription Kit (Qiagen, #205311, Germany). The resulting DNA and cDNA were amplified using the eukaryotic-specific V9 forward primer 1380F 5′-CCCTGCCHTTTGTACACAC-3′ and reverse primer 1510R 5′-CCTTCYGCAGGTTCACCTAC-3′ (Amaral-Zettler et al., 2009). The polymerase chain reaction (PCR) mixtures contained 12.5 μ*L* 2 × *Taq* PCR mix (Takara, China), ≤ 1 μg templates, 1 μ*L* forward primer (10 μ*M*), and 1 μ*L* reverse primer (10 μ*M*). RNase-free water was added to a final volume of 25 μ*L*. The PCR thermal cycle was performed under the following conditions: a denaturation step at 95°C for 5 min, 34 cycles of 94°C for 1 min, 57°C for 45 s, 72°C for 1 min, and a final extension at 72°C for 10 min. The duplicate PCR products were pooled and purified using a 2% agarose gel using QIAquick Gel Extraction Kit (Qiagen, #28704, Germany). All purified hypervariable V9 region amplicons were paired-end sequenced (2 × 250 bp) on the Illumina HiSeq 2500 platform at Novogene sequencing company (Beijing, China). Sequence data have been deposited in the NCBI SRA database under accession number SRP 151579.

### Sequence Processing and Statistical Analysis

Barcodes and primers were trimmed from paired-end sequences and then merged using FLASH (v. 1.2.7, http://ccb.jhu.edu/software/FLASH/; Magoč and Salzberg, 2011). A sequence quality check was performed using the Quantitative Insights Into Microbial Ecology (QIIME v. 1.7.0) pipeline (Caporaso et al., 2010). Chimeras were identified and removed with the chimera search module (USEARCH, version 4.2; Edgar et al., 2011) using the Silva 123 release (Quast et al., 2013) as a reference. The remaining high quality sequences were clustered into operational taxonomic units (OTUs) using a 95% similarity threshold with UPARSE (v. 7.0.100; Edgar, 2013). The representative sequence of each OTU (most abundant) was assigned using BLAST (Altschul et al., 1990) against the *V9_PR2* database (de Vargas et al., 2015). For further analysis, OTUs with only one read (singleton) as well as OTUs only represented in a single sample were discarded. operational taxonomic units (OTUs) only represented in DNA or RNA surveys were also removed from the downstream analyses, as biases in the rDNA copy number and chimeras resulted from the cDNA synthesis (Egge et al., 2013). operational taxonomic units (OTUs) assigned to Metazoan, Archaea, Bacteria, Organelle, and those that were unclassified were also excluded. To reduce the biases of sequencing coverage, the sequencing depth of the DNA and RNA results was separately rarefied to an equal sequence number based on their minimums.

Alpha diversity estimations (Shannon and Chao1) were calculated using QIIME. For the beta diversity, Bray-Curtis dissimilarity matrices were constructed based on the log-transformed relative abundance of each OTU using the “hclust” function in R (R Core Team., 2014). PERMANOVA was applied to establish the significant differences between dendrogram nodes resulting from the cluster analysis. Principal component analysis (PCA) was conducted to assess the difference in assemblage of picoeukaryotic communities among samples using the Vegan package in R (Oksanen et al., 2015). The RNA: DNA ratio of individual OTUs was calculated based on its proportions in the RNA and DNA results. Redundancy analysis (RDA) was performed using the Vegan package in R to identify the relationship between environmental parameters and the relative metabolic activities of invidious OTUs.

## Results

### Environmental Conditions of Sampling Stations

Stn. K2 and K3, located in the KC east area, were characterized by higher temperature and salinity (Table S1). The temperature ranged from 23.13 to 24.70°C and 19.78 to 20.24°C at the surface and DCM, respectively, while salinity ranged from 34.98 to 35.03 and 34.86 to 34.98 at the surface and DCM, respectively (Table S1). Stn. B1 and B2, located at the mixed area between KEC and OC (Figure 1), were characterized by lower temperature and salinity levels, with uniform distributions due to the vertical mixing of the water column (Table S1). The temperature ranged from 13.8 to 14.7°C and 13.74 to 14.33°C at the surface and DCM, respectively, while salinity ranged from 34.44 to 34.48 and 34.44 to 34.46 at the surface and DCM, respectively (Table S1). The Kuroshio front is defined by 15°C isotherms at a depth of 100 m (Kawamura et al., 1986). The vertical profile of the temperature and salinity across the B transect revealed that the KEC water and OC water were encountered near Stn. B3 (Figure S1). Maximum values of nutrient and Chl *a* concentrations were observed at the KEC and OC mixed water (Stn. B1 and B2, Table S1). At the DCM layer of Stn. B2, lower values of Chl *a* concentration were detected. Stn. A1a, A5, A8, and B9 were located around the KEC area and the nutrient concentrations at Stn. A1a (both at surface and DCM layers) and Stn. A5 (both at surface and DCM layers) as well as at the surface layer of Stn. A8 were all below detection (Table S1).

### Alpha Diversity of Picoeukaryotes

**Figure 2:**
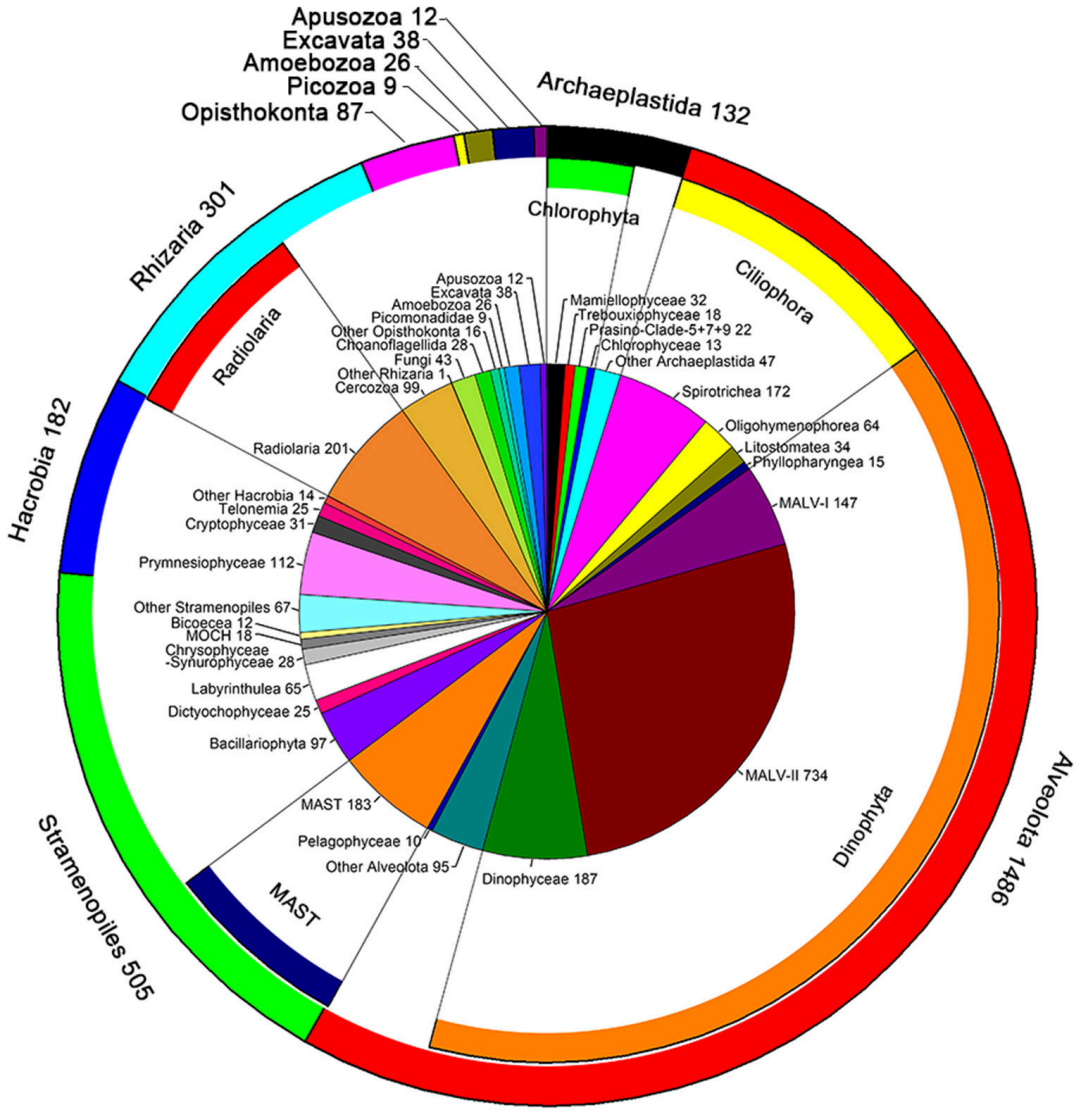
Number of OTUs unveiled by DNA and RNA surveys. The pie chart indicated the OTU numbers mainly at class level; the inner ring represented these classes belong to the phylum; the outer ring indicated the number of OTUs at super-group level.

A total of 661,340 DNA and 752,333 RNA sequences with an average length of 134 bp were generated and were clustered into 2,779 phylogenetically different OTUs both in the DNA and RNA datasets (95% similarity). The OTUs identified were affiliated to ten super-groups, including Archaeplastida, Alveolata, Stramenopiles, Hacrobia, Rhizaria, Opisthokonta, Picozoa, Amoebozoa, Excavata, and Apusozoa (Figure 2). Alveolata was the most diverse group, encompassing 1,486 OTUs. Most of them were affiliated to Dinophyta (1,117 OTUs) and Ciliophora (336 OTUs) (Figure 2). Within Dinophyta, marine alveolates (MALV)- II had the highest numbers of OTUs (734 OTUs), followed by Dinophyceae (187 OTUs) and MALV- I (147 OTUs). Within Ciliophora, Spirotrichea (172 OTUs), and Oligohymenophorea (64 OTUs) contributed largely followed by Litostomatea (34 OTUs) and Phyllopharyngea (15 OTUs). Stramenopiles was the second most diverse super-group, encompassing 505 OTUs, including members of the marine stramenopiles (MAST, 183 OTUs), Bacillariophyta (97 OTUs), Labyrinthulea (65 OTUs), Chrysophyceae-Synurophyceae (28 OTUs), Dictyochophyceae (25 OTUs), MOCH (18 OTUs), Bicoecea (12 OTUs), and Pelagophyceae (10 OTUs) (Figure 2). OTUs in Rhizaria (301 OTUs) were mainly affiliated to Radiolaria (201 OTUs) and Cercozoa (99 OTUs). The super-group Hacrobia (182 OTUs) was mainly composed of Prymnesiophyceae (112 OTUs), Cryptophyceae (31 OTUs) and Telonemia (25 OTUs) (Figure 2). One hundred and thirty two OTUs were affiliated to Archaeplastida, including members of Mamiellophyceae (32 OTUs), Trebouxiophyceae (18 OTUs), Prasino-Clade-5, −7, −9 (22 OTUs), and Chlorophyceae (13 OTUs). Members in Opisthokonta (87 OTUs), Picozoa (9 OTUs), Amoebozoa (26 OTUs), Excavata (38 OTUs), and Apusozoa (12 OTUs) made only minor contribution of the diversity of picoeukaryotes (Figure 2).

### Correlations Between Alpha Diversity and Environmental Factors

**Figure 3:**
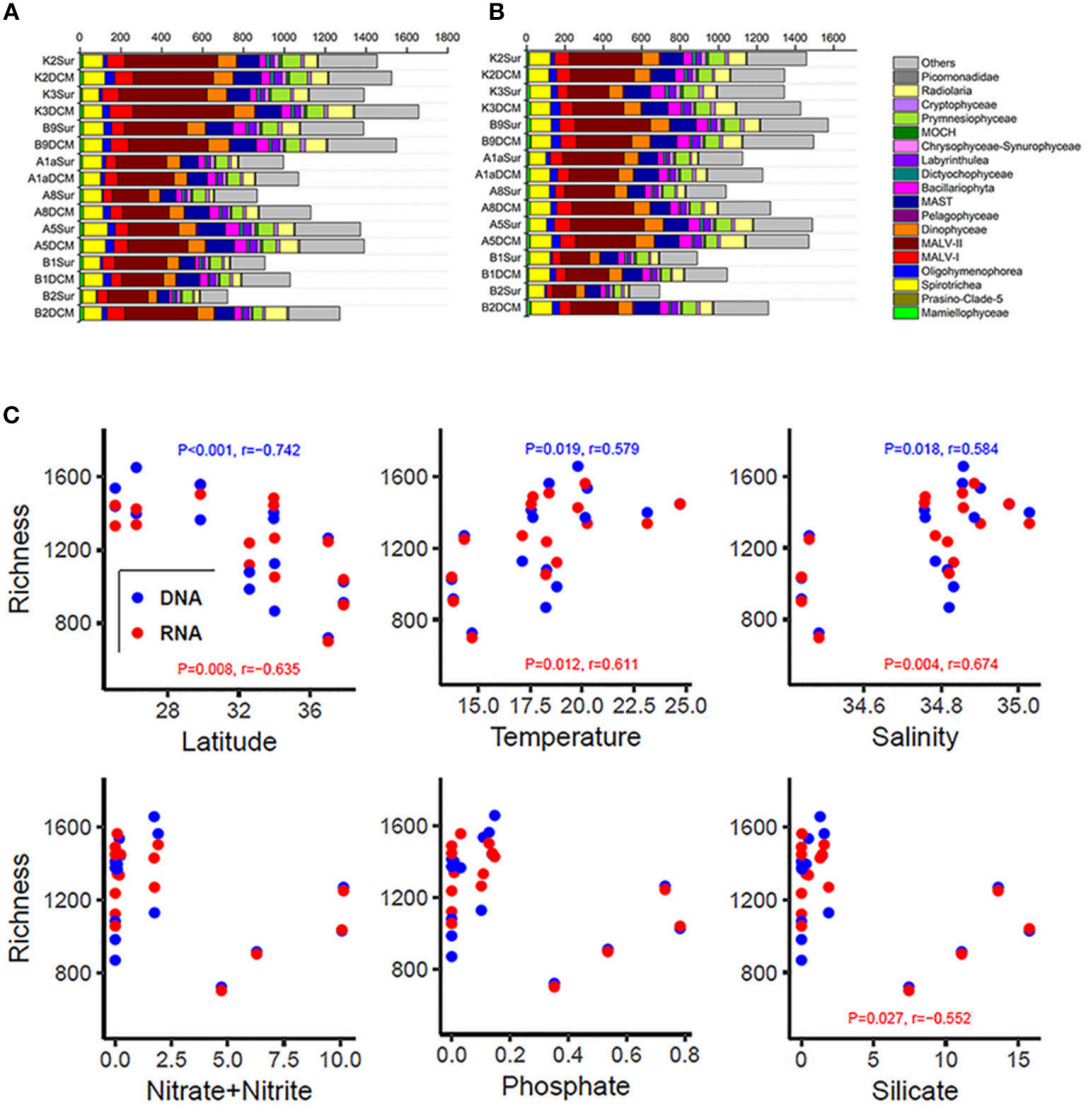
Number of OTUs of higher taxonomic groups (sequences proportion over 1% of the total DNA or RNA abundance) based on the DNA-derived **(A)** and RNA-derived **(B)** approaches for each sample. Others refer to groups with relatively lower abundance (sequences proportion < 1% of the total DNA and RNA abundance). **(C)** Pearson correlations between the number of OTUs and the latitude, temperature, salinity, nitrate + nitrite, phosphate, and silicate based on the DNA and RNA datasets at each sampling site.

The number of OTUs was varied substantially among samples (Figures 3A,B and Table S2). Higher number of OTUs was found in Stn. K2, K3, and B9 both in the DNA and RNA surveys (DNA: from 1,387 OTUs in B9Sur to 1,657 OTUs in K3DCM; RNA: from 1,342 in K3Sur to 1,571 in B9Sur), while lower values was found in the KEC and OC mixed water (Stn. B1 and B2) both in the DNA and RNA surveys (DNA: from 722 OTUs in the B2Sur to 1,028 OTUs in the B1DCM; RNA: from 692 OTUs in B2Sur to 1,044 OTUs in B1DCM), except for at the DCM layer of Stn. B2 (DNA: 1,270 OTUs; RNA: 1,260 OTUs) (Figures 3A,B and Table S2). The diversity indices, i.e., the Shannon and Chao 1 indices also showed the same trend in the DNA and RNA surveys (Table S2). MALV- II was always the most diverse group, varied from 18 to 31.22% in the individual DNA samples and from 15.95 to 25.25% in the individual RNA samples, respectively (Figures 3A,B). Statistical analysis revealed the number of OTUs in each sample inferred from the DNA and RNA approaches exhibited similar trends against the selected environmental factors (Figure 3C). The number of OTUs had a significantly negative relationship with latitude (DNA, *P* < 0.001, *r* = −0.742; RNA, *P* = 0.008, *r* = −0.635) and a significantly positive relationship with temperature (DNA, *P* = 0.019, *r* = 0.579; RNA, *P* = 0.012, *r* = 0.611) and salinity (DNA, *P* = 0.018, *r* = 0.584; RNA, *P* = 0.004, *r* = 0.674). The number of OTUs had no significant correlation with the nutrient concentrations, although the RNA-based OTU numbers in each sample was negatively correlated with SiO_3_ (Figure 3C)_._

### Community Composition of Picoeukaryotes

**Figure 4:**
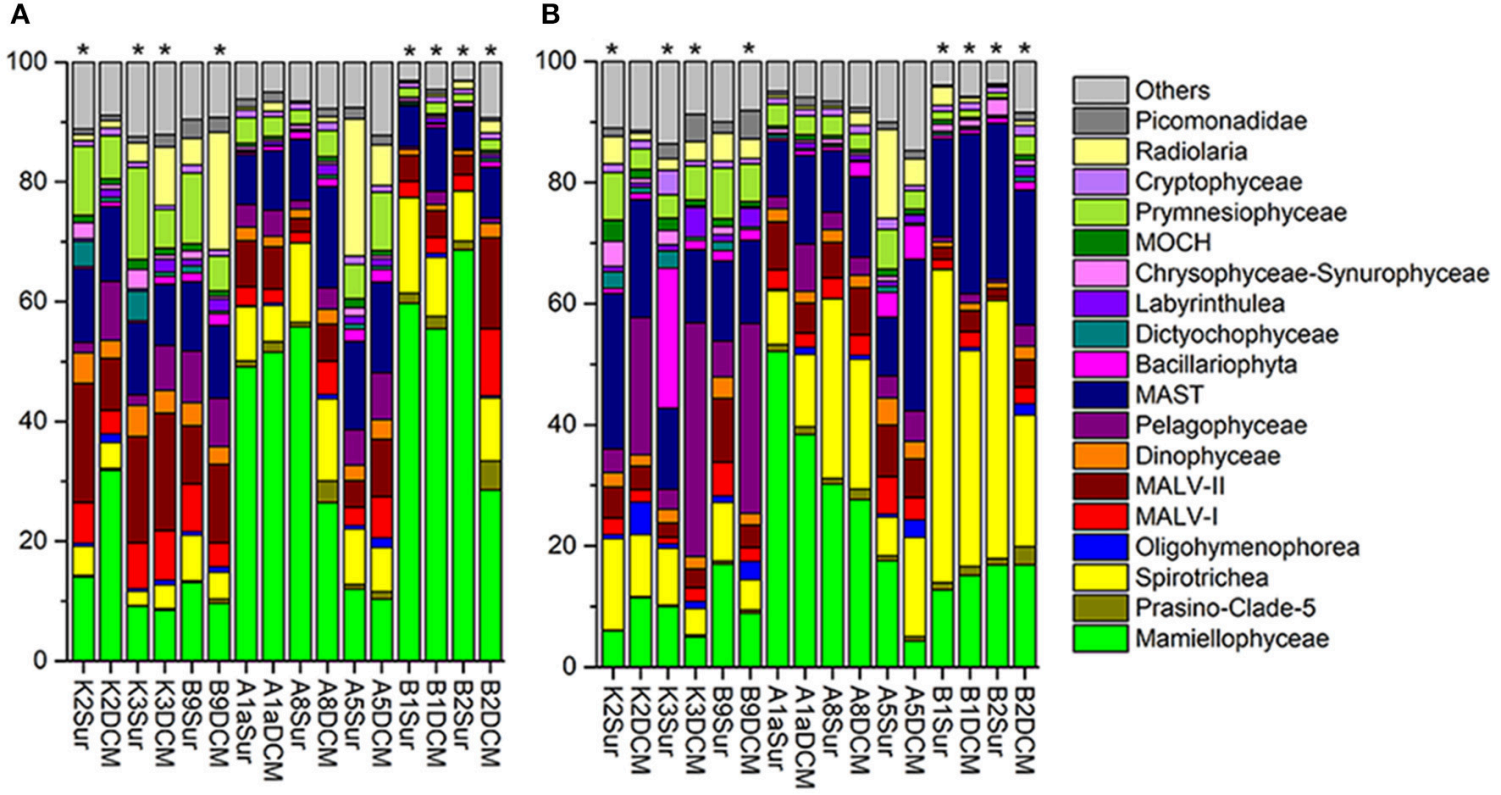
Relative abundance of higher taxonomic groups (sequences proportion over 1% of the total DNA or RNA abundance) based on the DNA-derived **(A)** and RNA-derived **(B)** approaches for each sample. Others refer to groups with relatively lower abundance (sequences proportion < 1% of the total DNA and RNA abundance). Asterisks indicate that the community composition was significantly different (*Chi-square test, P* < 0.05) between the DNA and RNA approaches for the same sample.

The number of the DNA and RNA reads ranged from 28,169 to 57,097 and from 36,483 to 57,507 per sample, respectively. To minimize the bias of the sequencing depth and allow for the comparison of sequencing results among samples, the DNA/RNA sequences were normalized to 28,169 (DNA) and 36,483 (RNA) in each sample, respectively (Table S2). The community captured by our DNA/RNA approaches were dominated by three super-groups, the Archaeplastida (33.54% of the total DNA reads; 20.05% of the total RNA reads), Alveolata (28.98% of the total DNA reads; 32.65% of the total RNA reads) and Stramenopiles (21.21% of the total DNA reads; 33.82% of the total RNA reads) (Figures S2A,B). Figures 4A,**B** showed the relative abundance of 18 dominant higher taxonomic groups (sequences proportion over 1% of the total DNA or RNA abundance) at each sample. Statistical analyses showed that the community composition inferred from the DNA and RNA approaches was significantly different from that of samples K2Sur, K3Sur, K3DCM, B9DCM, B1Sur, B1DCM, B2Sur, and B2DCM, with *P* < 0.05 (Figures 4A,B).

In the DNA survey, Mamiellophyceae, although little diverse (32 OTUs), was the most abundant group, representing 31.5% reads per sample on average (ranged from 8.46% reads in K3DCM to 68.63% reads in B2Sur) (Figures 2, 4A and Table 1). The most abundant OTU (24.22% of the total DNA reads) during our DNA survey was affiliated to *Ostreococcus lucimarinus* (OTU1, Table S4), which was observed with a peak of relative abundance in B2Sur (53.91%). MAST, mainly represented by MAST-4 (3.84%, on average) and MAST-3 (3.23%, on average), was the second abundant group, representing 11.13% reads per sample on average (Table 1). This group was less represented in the KEC and OC mixed water (Stn. B1 and B2, from 6.33% reads in B2Sur to 10.61% reads in B1DCM) and with higher relative abundance in A8DCM (16.89%) (Figure 4A). MALV-II was the most diverse group, representing 9.59% reads per sample on average (Figure 2 and Table 1). However, the relative abundance of its OTUs was detected always in low proportion. The most abundant OTU within MALV-II was affiliated to MALV-II-Clade-8_X sp. (OTU71, Table S4), which was only represented 0.35% reads per sample on average. Spirotrichea, the most diverse and abundant group in Ciliophora, was less represented in Stn. K2 and K3 (from 2.38% reads in K3Sur to 4.83% reads in K2Sur), and higher relative abundance was detected in Stn. A8 (13.34% reads in A8Sur and 13.73% in A8DCM) (Figures 2, 4A). Prymnesiophyceae, MALV-I and Radiolaria were detected in low proportion (< 6% reads per sample on average, Table 1), but occasionally reached higher proportions, for example, Prymnesiophyceae (11.81% reads in K3Sur), MALV-I (11.21% reads in B2DCM), and Radiolaria (22.92% reads in A5Sur) (Figure 4A). Only 10 OTUs in our survey was affiliated to Pelagophyceae, representing 3.94% reads per sample on average (Table 1). The most abundant OTU within this group was affiliated to *Pelagomonas calceolate* (OTU2, Table S4), which was detected with highest relative abundance in K2DCM (9.37%) (Figure 4A).

**Table 1:** The number of DNA and RNA sequences, and percentage of the total number of sequences by higher taxonomic groups (sequences proportion over 1% of the total DNA or RNA abundance).

**Higher taxonomic groups**	**DNA**		**RNA**	
	**Sequences**	**Percent**	**Sequences**	**Percent**
Mamiellophyceae	141,960	31.50	105,950	18.15
Prasino-Clade-5	5,937	1.32	5,497	0.94
Spirotrichea	36,862	8.18	109,432	18.75
Oligohymenophorea	2,678	0.59	7,385	1.27
MALV-I	22,679	5.03	16,588	2.84
MALV-II	43,244	9.59	29,892	5.12
Dinophyceae	12,099	2.68	12,648	2.17
Pelagophyceae	19,258	4.27	49,807	8.53
MAST	50,177	11.13	98,701	16.91
Bacillariophyta	4,555	1.01	17,243	2.95
Dictyochophyceae	4,649	1.03	5,042	0.86
Labyrinthulea	4,077	0.90	6,790	1.16
Chrysophyceae-Synurophyceae	4,245	0.94	6,726	1.15
MOCH	3,541	0.79	6,041	1.03
Prymnesiophyceae	26,623	5.91	23,326	4.00
Cryptophyceae	4,780	1.06	8,114	1.39
Radiolaria	21,433	4.76	16,903	2.90
Picomonadidae	5,615	1.25	8,590	1.47
Others	36,292	8.05	49,053	8.40

*The other category refers to groups with relatively lower abundance (sequences proportion < 1% of the total DNA and RNA abundance)*.

In the RNA survey, Spirotrichea was the most abundant group, representing 18.75% reads per sample on average (Figure 4B and Table 1). This group was highly abundant in the KEC and OC mixed water (Stn. B1 and B2), reaching up to 51.64% of the reads in B1Sur (Figure 4B). Within Spirotrichea, the most abundant OTU was affiliated to s*trobilidiidae_X* sp. (OTU3, Table S4), was also highly represented in B1Sur (31.14%). In general, the relative abundance of Mamiellophyceae in the RNA samples (18.15%, on average) was lower than in the DNA samples (Figures 4A,B and Table 1). OTU1 (*O. lucimarinus*) was also the most abundant OTU during the RNA survey (Table S4), with the highest relative abundance reaching up to 41.37% of the reads in A1aSur. MAST represented 16.91% of the reads per sample on average (Table 1), and was mainly dominated by MAST-4 (10.18%, on average). High contributions of MAST-4 reads were detected in the KEC and OC mixed water (Stn. B1 and B2), ranging from 6.75% reads in B1Sur to 10.23% reads to B2DCM. MAST-3 represented 5.66% reads per sample on average, and was occasionally highly abundant, reaching 21.43% of the reads in A5DCM. Pelagophyceae (8.53%, on average, Table 1) was dominant by *P. calceolate* (OTU2, Table S4), which was highly abundant at the DCM layers of Stn. K2, K3, and B9 (Figure 4B). MALV-II represented 5.12% reads per sample on average (Table 1). The most abundant OTU (MALV-II_XX sp., OTU58, Table S4) within this group was detected in rather low proportion (0.18%, on average). Bacillariophyta (2.95%, on average) and Radiolaria (2.9%, on average) were less abundant, but occasionally detected with higher proportions, for example, Bacillariophyta (29.92% reads in K3Sur), Radiolaria (14.73% reads in A5Sur) (Figure 4B and Table 1).

Based on the HPLC method, phytoplankton communities were characterized by a relatively high dominance of Prasinophytes and Haptophytes_8 (Figure S3A). The contribution of *Prochlorococcus* and *Synechococcus* was decreased with the increasing latitude, and the contribution of diatoms was highest in samples collected at Stn. A5 (Figure S3A). Cell abundance of Picoeukaryotes estimated by flow cytometry was generally increased with the increasing latitude, while low values were detected in samples A8DCM, A5Sur, A5DCM, and B2DCM (Figure S3B).

### Beta Diversity of Picoeukaryotic Assemblages in the Northwestern Pacific Ocean

**Figure 5:**
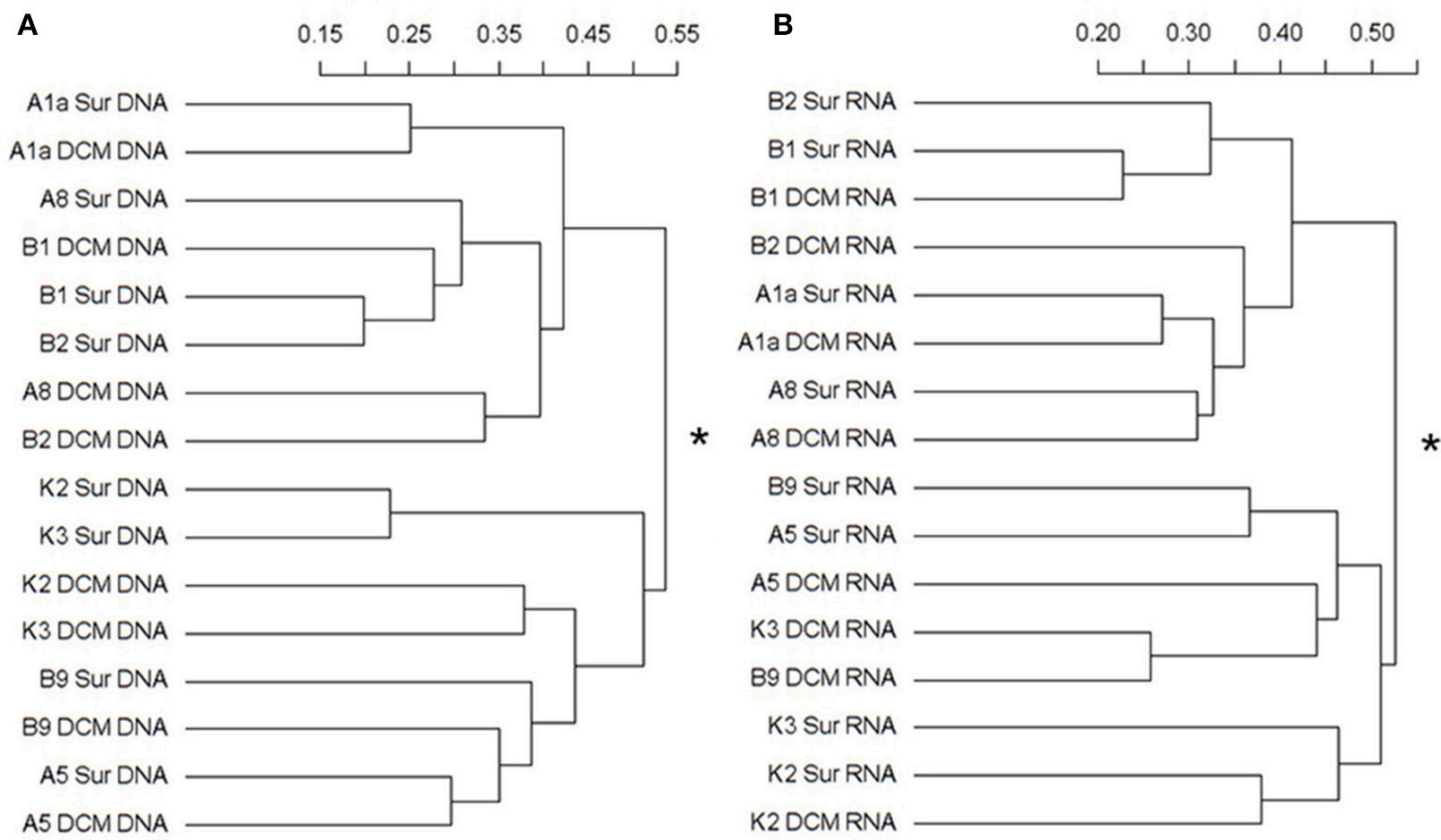
Cluster diagram of the Bray-Curtis dissimilarities based on the log-transformed relative OTU abundance for each sample. The separated DNA **(A)** and RNA **(B)** community groups (with asterisks at the node) exist with significant compositional differences (DNA: *r*^2^ = 0.31718, *P* = 0.001; RNA: *r*^2^ = 0.27023, *P* = 0.001) as determined by PERMANOVA. The percent dissimilarity among samples is shown along the horizontal axes.

The Bray-Curtis dissimilarity results from the DNA and RNA sequences were both clustered the samples into two groups (Figures 5A,B). In the DNA survey, the community composition of Stn. A1a, A8, B1, and B2, where Mamiellophyceae was the dominant group, was significantly different from Stn. K2, K3, and B9 according to the statistical analyses (PERMANOVA, *r*^2^ = 0.31718, *P* = 0.001) (Figure 5A). The samples collected in the KEC and OC mixed water (Stn. B1 and B2) were grouped together, except for the sample B2DCM, which was grouped as a sister cluster with A8DCM (Figure 5A). In Stn. A1a and A5, the samples collected at the surface and DCM layers were grouped as sister clusters (Figure 5A), indicating the minor composition difference in the two depths at these two stations. In the RNA survey, the samples were clustered with a similar pattern as the results of the DNA analysis, and the community composition of the two clusters was also significantly different (PERMANOVA, *r*^2^ = 0.27023, *P* = 0.001) (Figure 5B). The sample collected at the DCM layer of Stn. B2 was grouped separately as a single cluster (Figure 5B). Minor differences in the community composition at the surface and DCM layers were observed in Stn. B1 and A1a (Figure 5B).

### Comparison Between DNA- and RNA-Based Picoeukaryotic Communities

**Figure 6:**
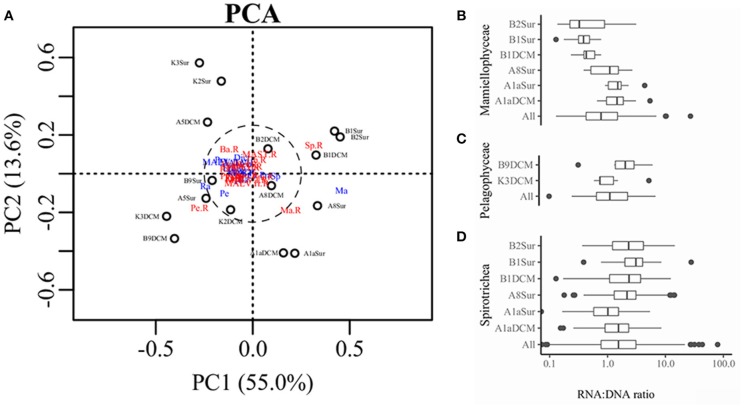
**(A)** Scatter diagram of PC_1_ and PC_2_ derived from the relative abundance of higher taxonomic groups in the DNA and RNA databases detected in each sample, blue color indicated the sequences was derived from the DNA survey and red color indicated the sequences was derived from the RNA survey. Ma, Mamiellophyceae; Sp, Spirotrichea; Pe, Pelagophyceae; Ba, Bacillariophyta; Dict, Dictyochophyceae; Prym, Prymnesiophyceae. Boxplot representing the RNA: DNA ratios for Mamiellophyceae **(B)**, Pelagophyceae **(C)**, and Spirotrichea **(D)** in all and individual samples.

**Figure 7:**
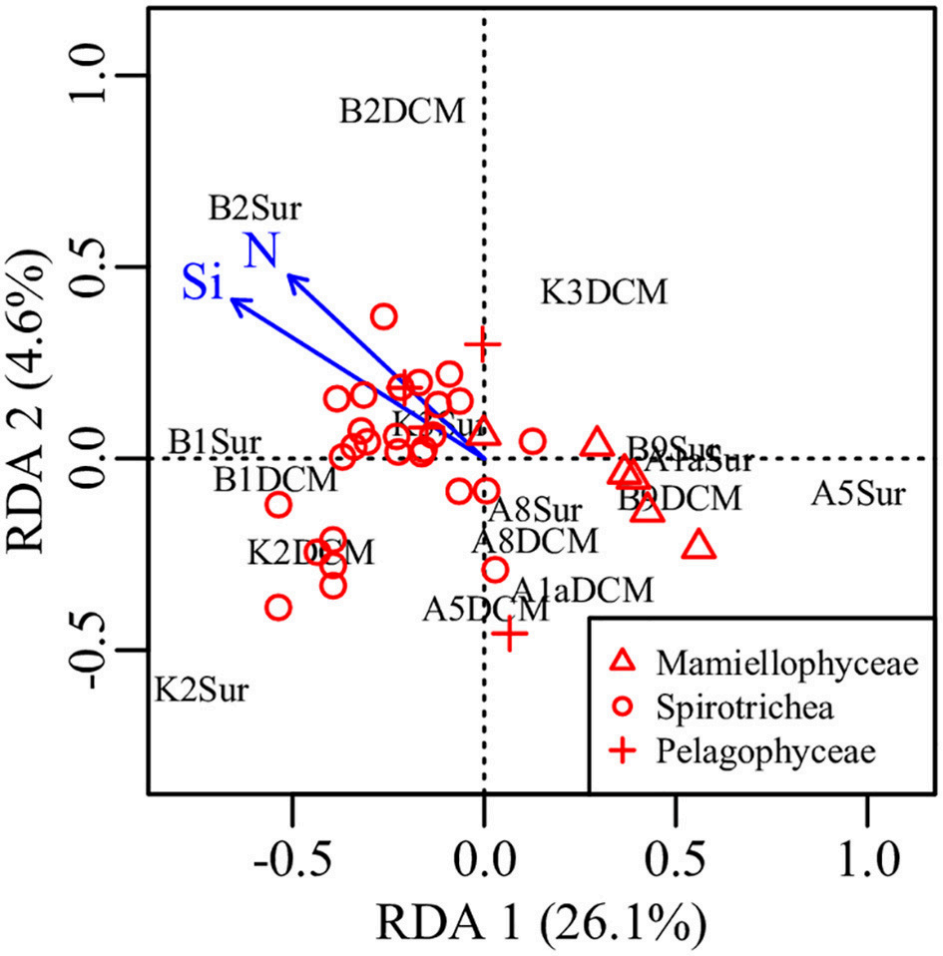
Plot of the RDA integrating environmental factors and the relative metabolic activities of Mamiellophyceae, Pelagophyceae, and Spirotrichea species. N, nitrate + nitrite, Si, silicate.

PCA was conducted on the variabilities of the higher taxonomic groups among samples (Figure 6A). The top variables contributed to the total variabilities were Mamiellophyceae DNA and RNA sequences, Spirotrichea RNA sequences, and Pelagophyceae RNA sequences (Figure 6A). According to the scatter plot of first two axes, these four variables illustrated several sampling stations and depths with distinct picoeukaryotic composition: B1Sur, B2Sur, and B1DCM were associated with relative high abundance of Spirotrichea RNA sequences and Mamiellophyceae DNA sequences, but lesser Mamiellophyceae RNA sequences; by contrast, A8Sur, A1aSur, and A1aDCM were related with relative high abundance of both DNA and RNA sequences of Mamiellophyceae, but lesser Spirotrichea RNA sequences; meanwhile, K3DCM and B9DCM showed relative high Pelagophyceae RNA sequences (Figure 6A). The Figures 4A,B displayed these features as well. Taking all samples together, the RNA: DNA ratio (as a proxy of cell relative metabolic activity) for Mamiellophyceae (average RNA: DNA ratio = 1.38) was slightly above 1. Varied ratios was observed in individual samples, with higher values was found in the samples A8Sur, A1aSur, and A1aDCM and lower values was found in samples B1Sur, B1DCM, and B2Sur (Figure 6B). High relative metabolic activity of Pelagophyceae (average RNA: DNA ratio = 1.64) was recorded in the sample B9CM (Figure 6C). High relative metabolic activity of Spirotrichea (average RNA: DNA ratio = 2.79) was recorded in the samples B1Sur, B1DCM, B2Sur, and A8Sur (Figure 6D). At the OTU level, the species within these three groups presented varied DNA: RNA ratio across samples (Figure S4 and Table S3), indicating their changing relative cell activity in different environmental conditions in the northwestern Pacific Ocean. Redundancy analysis (RDA) showed this varied cell activities was significantly affected by the NO_3_ + NO_2_ and SiO_3_ concentration (Figure 7).

## Discussion

Previously, most studies of picoeukaryotic diversity were mainly based on the rDNA analysis (Vaulot et al., 2008; Lepère et al., 2009; Shi et al., 2009; Wu et al., 2014). Though, DNA-based survey allows for the investigation of all present picoeukaryotic species, this method could lead several biases by including DNA from the dissolved extracellular pools or dormant/dead cells (Karl and Bailiff, 1989). The RNA-based molecular survey better depict picoeukaryotic diversity and indicate the active fractions in natural sample, although this approach faced a numbers of limitations (Blazewicz et al., 2013). Overall, combined the DNA with RNA molecular surveys provide complementary information for picoeukaryotic diversity in a natural environment. First, picoeukaryotic diversity was less studied in the northwestern Pacific Ocean. The current study used HTS based on the DNA and RNA sequences to access the picoeukaryotic diversity and assemblages. Second, the northwestern Pacific Ocean is characterized by intricate oceanic currents and complex oceanographic conditions. Information on how picoeukaryotic diversity and relative metabolic activities impacted by environmental factors in this area is rare. In the present study, the correlation between picoeukaryotic diversity, relative metabolic activities and environmental factors were conducted.

### Correlations Between Picoeukaryotic Diversity and Environmental Factors in the Northwestern Pacific Ocean

Previous studies that investigated the relationships between picoeukaryotic assemblages and environmental factors have shown that these micro-organisms respond to many factors (Not et al., 2005; Hamilton et al., 2008; Kirkham et al., 2013; Bellaaj Zouari et al., 2018). In the Gulf of Gabès, picoeukaryotic diversity was known to be affected by both physical and chemical factors (Bellaaj Zouari et al., 2018). In the Atlantic-Arctic confluence region, picoeukaryotic assemblages was affected by geographic proximity, abiotic, and biotic factors and water mass origin (Hamilton et al., 2008). Nutrient concentration was also an important environmental factor affecting the picoeukaryotic assemblages, e.g., the distribution pattern of Prymnesiophyceae and Chrysophyceae was possibly associated with the nitrogen: phosphorus (N: P) ratio (Kirkham et al., 2013) and *micromonas pusilla* was more likely abundant in nutrient rich environments (Not et al., 2005). In the present study, picoeukaryotic diversity was positively correlated with temperature and salinity (Figure 3C), indicating that the two environmental factors played an important role in affecting the diversity of picoeukaryotes in the northwestern Pacific Ocean. Picoeukaryotic diversity appears not to be correlated with the nutrient concentrations; however, the number of OTUs was decreased with the increasing nutrient concentrations. High values of the numbers of OTUs were occurred at the stations with low nutrient concentrations, while lower values were detected in the converse environmental conditions (Figure 3C).

### Picoeukaryotic Assemblages in the Northwestern Pacific Ocean

Picoeukaryotes in the northwestern Pacific Ocean were mainly represented by Mamiellophyceae, MAST, MALV-II, Spirotrichea, Prymnesiophyceae, and MALV-I (69.33% of the total DNA reads) in the DNA survey, and by Spirotrichea, Mamiellophyceae, MAST, Pelagophyceae, and MALV-II (67.46% of the total RNA reads) in the RNA survey (Table 1 and Figures 4A,**B**). The large proportion of Mamiellophyceae was contributed by *O. lucimarinus* (OTU1, Table S4) both in the DNA and RNA surveys. This species is surface and high-light-adapted ecotype, and have been found in coastal and marine environments (Palenik et al., 2007; Demir-Hilton et al., 2011). The decreased proportion of Mamiellophyceae in the RNA survey compared to its DNA counterpart, particularly in the KEC and OC mixed water samples (Stn. B1 and B2) (Figures 4A,B), suggested that part of Mamiellophyceae DNA molecular during our survey may obtained from dormant or dead cells. The contribution of prasinophytes based on our HPLC method showed comparable pattern of Mamiellophyceae assemblages based on the DNA survey across samples (Figure S3A and Figure 4A). The calculation of pigment-based composition was largely influenced by the increasing accessory pigment to chl *a* ratios from lower latitude to higher latitude by photo-acclimation. The CHEMTAX calculation might overestimate the percentage of Prasinophytes at Stn. B1 and B2, however the mechanism was different from that of more relative abundance of DNA than RNA. The high proportion of prasinophytes in the KE and adjacent regions was consistent with a recent study reporting prasinophytes became a major group of phytoplankton during the spring bloom (Nishibe et al., 2017). MALV-I and MALV-II, most of which were characterized as parasites on a range of hosts (Skovgaard et al., 2005; Bachvaroff et al., 2012), have been shown to be abundant groups of picoeukaryotes based on the DNA survey in different marine ecosystems (Guillou et al., 2008; Massana, 2011), while were less represented in the RNA survey (Massana et al., 2015). The higher genomic rDNA copy numbers than other picoeukaryotes is the most reasonable explanation for this discrepancy (Massana et al., 2015). During our survey, the overrepresented of the DNA sequences of MALV-I, -II than their RNA counterparts was also observed (Figures 4A,B and Table 1). *P. calceolate*, the dominant OTU in Pelagophyceae (Table S4), is a low-light-adapted species with the ability to adapt well to environmental conditions in the DCM layer (Timmermans et al., 2005; Shi et al., 2009). In our survey, higher proportion of *P. calceolate* sequences in the DCM layer than the surface layer was also observed both in the DNA and RNA datasets (Table S4).

In the Bray-Curtis dissimilarity analysis, samples were clustered into two groups both in the DNA and RNA surveys (Figures 5A,B). Samples collected at Stn. B1, B2, A1a, A8 were grouped separate from samples collected at Stn. B9, A5, K3, K2, suggesting the presence of a significant compositional differences in these two clusters. Notably, the samples (DNA/RNA) collected at the DCM layer of Stn. B2 were always clustered separately from the other samples collected in the Stn. B1 and B2 (Figures 5A,B), and the number of OTUs, Chl *a* concentration and FCM data were also very different from the other samples in Stn. B1 and B2 (Figures 3A,B, Table S1, and Figure S3B). These differences appear to cause by the complex horizontal and vertical motion of seawater in the KEC and OC mixed water.

### Relative Metabolic Activities of Picoeukaryotes in the Northwestern Pacific Ocean

Nowadays, it is generally accepted that protist assemblages inferred from DNA survey represented the whole species, whereas its RNA counterparts specifically represented the active fraction (Massana et al., 2015), in spite of several limitations should be carefully considered in this approach, e.g., the dormant or dead cells (Stoeck et al., 2007; Not et al., 2009), the varied gene copy numbers (Godhe et al., 2008; Gong et al., 2013) and cell size (Zhu et al., 2005). In the present study, picoeukaryotic community composition inferred from DNA and RNA approaches was significantly different (Figures 4A,B), and the top variables contributed to the total variabilities were the sequences (DNA or RNA) affiliated to Mamiellophyceae, Pelagophyceae, and Spirotrichea (Figure 6A). Previous studies have used the RNA: DNA ratio as a proxy for the relative metabolic activity of taxonomic groups of protist (Hu et al., 2016; Xu et al., 2017). However, the varied RNA: DNA ratios of taxonomic groups among samples should be interpreted cautiously, because the presence of rRNA indicates the possible ribosomal activity and the potential of protein synthesis, instead of providing an indicator of cell activity (Blazewicz et al., 2013). Confined to a specific group, Wu and Liu (2018) found the relative metabolic activities of Bacillariophyceae species in Pearl River-South China Sea Continuum were significantly affected by the PO_4_ concentration and salinity. In order to obtain continuous relationship of the relative metabolic activities of individual OTUs in our samples, we selected OTUs only occurred in both DNA and RNA surveys (Hu et al., 2016) and appeared in all samples within Mamiellophyceae, Pelagophyceae, and Spirotrichea for the further metabolic activities analyses. The varied RNA: DNA ratios of individual OTUs across samples suggested the changing metabolic activities of these species in varied environmental conditions (Figure S4 and Table S3), and the relative metabolic activities of these species was mainly affected by the nutrient concentration, i.e., the NO_3_ + NO_2_ and SiO_3_ concentration (Figure 7).

In the KEC and OC mixed water samples (Stn. B1 and B2), the relative activities of Mamiellophyceae species were stayed at a low level, while the Spirotrichea species were highly active (Figure S4 and Table S3). Spirotrichea was found to be an active predator of picoeukaryotes and bacteria in the euphotic zone (Calbet and Landry, 2004; McKie-Krisberg and Sanders, 2014). Moreover, MAST-4, which was potential predator of picoeukaryotes (Lin et al., 2012), was also found very active in the KEC and OC mixed water (Stn. B1 and B2) in our RNA survey (Figure 4B). Dilution experiments in the same cruise revealed that there was no significant difference in the food selection for microzooplankton in the KEC and OC mixed water. Thus, the abundant picoplankton were more likely to be grazed (unpublished data). So, we suggested that Mamiellophyceae species probably be grazed during our survey, and the overrepresented DNA sequences in the KEC and OC mixed water samples (Stn. B1 and B2) may partially contributed by the dead materials.

## Concluding Remarks

This study represents the first contribution regarding the diversity and activity of picoeukaryotes in the northwestern Pacific Ocean based on the HTS (DNA/RNA). Our finding showed that picoeukaryotes exists high and variable diversity in the study area with different hydrologic conditions. Temperature and salinity were the two important environmental factors affecting the picoeukaryotic diversity. The relative metabolic activities of Mamiellophyceae, Spirotrichea, and Pelagophyceae species were highly affected by the nutrient concentration, i.e., the NO_3_ + NO_2_ and SiO_3_ concentration. Due to the lack of sufficient samples in our survey and the complex oceanographic conditions in the northwestern Pacific Ocean, further investigations are needed to accumulate sufficient data during seasons and years. Overall, these findings contributed to the understanding of picoeukaryotic assemblages and activity in the open ocean system.

## Author Contributions

FW, BH, and LW designed the experiments and onboarded the processing. FW carried out the experiment and wrote the paper. FW, YX, WW, and PS analyzed the data and improved the manuscript.

### Conflict of Interest Statement

The authors declare that the research was conducted in the absence of any commercial or financial relationships that could be construed as a potential conflict of interest.
