# Supplementary material for: Picoeukaryotic Diversity And Activity in the Northwestern Pacific Ocean Based on rDNA and rRNA High-Throughput Sequencing
Source: Front Microbiol. 2019 Jan 9;9:3259. doi: 10.3389/fmicb.2018.03259 (PMC6333705; doi:10.3389/fmicb.2018.03259)
Supplement: Supplementary file 1 [file Data_Sheet_1.docx]

***Supplementary Material***

**Picoeukaryotic diversity and activity** **in the northwestern Pacific Ocean based on rDNA and rRNA high-throughput sequencing**

**Feipeng Wang, Yuyuan Xie, Wenxue Wu, Ping Sun, Lei Wang, Bangqin Huang***

*** Correspondence:** Bangqin Huang: [bqhuang@xmu.edu.cn](mailto:bqhuang@xmu.edu.cn)

# Supplementary Tables

**Table S1.** Environmental conditions in the sampling stations in the northwestern Pacific Ocean during the spring cruise in 2015. BDL means nutrient concentration below detection limit.

| Stations | Date | Longitude | Latitude | Layer (m) | Temperature (°C) | Salinity (psu) | PO_4_ (μM) | SiO_3_ (μM) | NO_3_ + NO_2_ (μM) | Chl *a* (μg L^-1^) |
| --- | --- | --- | --- | --- | --- | --- | --- | --- | --- | --- |
| K2 | Apr-5 | 134.00 | 25.03 | 5 | 24.70 | 34.98 | 0.14 | 1.45 | 0.23 | 0.08 |
| K2 | Apr-5 | 134.00 | 25.03 | 75 | 20.24 | 34.90 | 0.11 | 0.50 | 0.18 | 0.30 |
| K3 | Apr-7 | 136.65 | 26.21 | 5 | 23.13 | 35.03 | 0.01 | 0.32 | 0.09 | 0.14 |
| K3 | Apr-7 | 136.65 | 26.21 | 75 | 19.78 | 34.86 | 0.15 | 1.31 | 1.73 | 0.26 |
| B9 | Apr-10 | 146.88 | 29.82 | 5 | 20.14 | 34.88 | 0.03 | 0.05 | 0.09 | 0.06 |
| B9 | Apr-10 | 146.88 | 29.82 | 60 | 18.40 | 34.86 | 0.13 | 1.57 | 1.90 | 0.56 |
| A1a | Apr-14 | 145.95 | 32.59 | 5 | 18.79 | 34.83 | BDL | BDL | BDL | 0.11 |
| A1a | Apr-14 | 145.95 | 32.59 | 50 | 18.30 | 34.82 | BDL | BDL | BDL | 0.12 |
| A8 | Apr-21 | 152.00 | 34.00 | 5 | 18.26 | 34.82 | BDL | BDL | BDL | 0.07 |
| A8 | Apr-21 | 152.00 | 34.00 | 65 | 17.12 | 34.78 | 0.10 | 1.91 | 1.74 | 0.10 |
| A5 | Apr-23 | 149.03 | 33.95 | 5 | 17.62 | 34.76 | BDL | BDL | BDL | 0.10 |
| A5 | Apr-23 | 149.03 | 33.95 | 50 | 17.54 | 34.76 | BDL | BDL | BDL | 0.07 |
| B1 | Apr-26 | 147.02 | 37.88 | 5 | 13.80 | 34.44 | 0.54 | 11.09 | 6.29 | 0.17 |
| B1 | Apr-26 | 147.02 | 37.88 | 30 | 13.74 | 34.44 | 0.78 | 15.78 | 10.07 | 0.04 |
| B2 | Apr-27 | 147.02 | 37.03 | 5 | 14.70 | 34.48 | 0.35 | 7.45 | 4.73 | 0.23 |
| B2 | Apr-27 | 147.02 | 37.03 | 25 | 14.33 | 34.46 | 0.73 | 13.63 | 10.14 | 0.05 |

**Table S2.** Sequences information (number of raw sequences and after quality-check, chimera removed), OTU information (number of OTUs and the OTU numbers after removed singletons, DNA-only and RNA-only and after normalizing), and diversity index (Shannon index and Chao 1 index) across samples.

| Sample | Raw reads after combining | Quality-check reads | Chimera-removed reads | Number of OTU | Numbers of OTU without singletons and DNA-only and RNA-only OTU | Number of picoeukaryotic OTUs (without metazoa) | Numbers of picoeukaryotic OTUs after normalizing (DNA:28,169 reads, RNA:36483 reads) | Shannon index | Chao1 Index |
| --- | --- | --- | --- | --- | --- | --- | --- | --- | --- |
| K2 Sur DNA | 54,024 | 46,405 | 46,067 | 1,933 | 1,704 | 1,621 | 1,441 | 8.20 | 1,900 |
| K2 Sur RNA | 63,307 | 61,395 | 60,854 | 1,961 | 1,768 | 1,683 | 1,444 | 7.81 | 2,137 |
| K2 DCM DNA | 50,298 | 40,406 | 40,024 | 2,012 | 1,835 | 1,754 | 1,535 | 6.90 | 2,127 |
| K2 DCM RNA | 48,175 | 48,110 | 47,468 | 1,739 | 1,618 | 1,532 | 1,332 | 6.73 | 2,027 |
| K3 Sur DNA | 57,906 | 46,056 | 45,805 | 1,914 | 1,707 | 1,615 | 1,396 | 8.18 | 1,990 |
| K3 Sur RNA | 59,559 | 58,263 | 57,978 | 1,911 | 1,655 | 1,548 | 1,337 | 7.64 | 2,000 |
| K3 DCM DNA | 49,271 | 40,016 | 39,666 | 22,58 | 1,943 | 1,852 | 1,653 | 8.44 | 2,245 |
| K3 DCM RNA | 62,868 | 62,669 | 62,161 | 1,928 | 1,775 | 1,667 | 1,425 | 5.86 | 2,111 |
| B9 Sur DNA | 49,791 | 47,448 | 47,219 | 1,804 | 1,695 | 1,593 | 1,367 | 7.64 | 2,033 |
| B9 Sur RNA | 57,430 | 51,215 | 50,915 | 1,998 | 1,875 | 1,772 | 1,557 | 7.98 | 2,226 |
| B9 DCM DNA | 53,439 | 53,294 | 52,912 | 2,087 | 1,893 | 1,790 | 1,561 | 7.64 | 2,160 |
| B9 DCM RNA | 63,144 | 55,797 | 55,404 | 1,960 | 1,814 | 1,723 | 1,502 | 6.40 | 2,145 |
| A1a Sur DNA | 63,318 | 61,029 | 60,766 | 1,321 | 1,273 | 1,197 | 982 | 5.43 | 1,550 |
| A1a Sur RNA | 57,040 | 56,916 | 56,696 | 1,445 | 1,376 | 1,288 | 1,117 | 5.43 | 1,591 |
| A1a DCM DNA | 49,892 | 47,705 | 47,430 | 1,424 | 1,358 | 1,283 | 1,078 | 5.17 | 1,657 |
| A1a DCM RNA | 57,629 | 53,363 | 52,807 | 1,598 | 1,504 | 1,412 | 1,236 | 6.05 | 1,836 |
| A8 Sur DNA | 56,875 | 56,708 | 56,565 | 1,118 | 958 | 866 | 866 | 4.55 | 1,218 |
| A8 Sur RNA | 56,480 | 56,339 | 56,053 | 1,353 | 1,293 | 1,197 | 1,053 | 6.21 | 1,527 |
| A8 DCM DNA | 59,107 | 57,418 | 56,930 | 1,453 | 1,372 | 1,294 | 1,125 | 6.95 | 1,781 |
| A8 DCM RNA | 52,230 | 49,113 | 48,685 | 1,596 | 1,514 | 1,429 | 1,265 | 6.87 | 1,852 |
| A5 Sur DNA | 57,265 | 53,548 | 53,243 | 1,830 | 1,708 | 1,619 | 1,370 | 6.97 | 2,157 |
| A5 Sur RNA | 51,257 | 44,142 | 43,862 | 1,661 | 1,561 | 1,487 | 1,487 | 7.33 | 2,125 |
| A5 DCM DNA | 52,733 | 44,310 | 44,096 | 1,828 | 1,703 | 1,609 | 1,408 | 7.67 | 2,017 |
| A5 DCM RNA | 51,069 | 50,955 | 50,693 | 1,894 | 1,734 | 1,636 | 1,447 | 7.51 | 2,109 |
| B1 Sur DNA | 59,596 | 56,515 | 56,284 | 1,262 | 1,211 | 1,143 | 912 | 4.38 | 1,639 |
| B1 Sur RNA | 53,091 | 52,971 | 52,684 | 1,188 | 1,147 | 1,068 | 898 | 5.42 | 1,495 |
| B1 DCM DNA | 49,254 | 45,034 | 44,792 | 1,373 | 1,319 | 1,249 | 1,026 | 5.04 | 1,620 |
| B1 DCM RNA | 61,485 | 57,567 | 57,157 | 1,378 | 1,308 | 1,233 | 1,036 | 6.21 | 1,681 |
| B2 Sur DNA | 64,016 | 63,853 | 63,719 | 1,150 | 1,093 | 1,016 | 721 | 3.89 | 1,474 |
| B2 Sur RNA | 51,366 | 51,262 | 51,047 | 952 | 897 | 825 | 697 | 4.86 | 1,172 |
| B2 DCM DNA | 54,836 | 45,478 | 45,219 | 1,711 | 1,559 | 1,478 | 1,264 | 6.70 | 1,919 |
| B2 DCM RNA | 65,644 | 65,486 | 64,652 | 1,670 | 1,563 | 1,466 | 1,245 | 7.06 | 1,902 |

**Table S3.** RNA: DNA ratios of OTUs occurred in both DNA and RNA surveys and recorded in all samples within Mamiellophyceae, Pelagophyceae and Spirotrichea.

| OTU | Affiliation | K2Sur | K2DCM | K3Sur | K3DCM | B9Sur | B9DCM | A1aSur | A1aDCM | A8Sur | A8DCM | A5Sur | A5DCM | B1Sur | B1DCM | B2Sur | B2DCM |
| --- | --- | --- | --- | --- | --- | --- | --- | --- | --- | --- | --- | --- | --- | --- | --- | --- | --- |
| OTU_1 | *Ostreococcus lucimarinus* | 0.43 | 0.35 | 0.96 | 0.52 | 1.39 | 0.84 | 1.03 | 0.66 | 0.44 | 1.10 | 1.35 | 0.35 | 0.18 | 0.23 | 0.24 | 0.61 |
| OTU_7 | *Bathycoccus prasinos* | 0.46 | 0.40 | 0.52 | 0.68 | 1.45 | 1.11 | 1.46 | 1.71 | 0.81 | 0.76 | 1.67 | 0.56 | 0.39 | 0.42 | 0.28 | 0.54 |
| OTU_9 | *Micromonas pusilla* | 0.32 | 0.37 | 0.75 | 0.97 | 0.77 | 0.75 | 0.91 | 0.88 | 1.74 | 1.29 | 2.57 | 0.53 | 0.33 | 0.40 | 0.38 | 0.89 |
| OTU_13 | *Micromonas* sp. | 0.41 | 0.34 | 1.81 | 0.65 | 1.55 | 1.81 | 1.56 | 1.93 | 1.42 | 0.96 | 1.91 | 0.63 | 0.31 | 0.36 | 0.19 | 0.31 |
| OTU_43 | *Micromonas* Clade B4 | 0.63 | 0.46 | 10.26 | 0.81 | 0.77 | 0.90 | 1.02 | 1.24 | 2.68 | 1.45 | 0.56 | 0.48 | 0.38 | 0.43 | 3.12 | 4.49 |
| OTU_54 | *\|Micromonas pusilla* | 0.36 | 0.37 | 0.32 | 0.41 | 4.30 | 1.00 | 2.30 | 1.38 | 1.51 | 0.85 | 3.45 | 0.74 | 0.42 | 0.56 | 0.14 | 0.44 |
| OTU_2 | *Pelagomonas calceolata* | 2.11 | 2.30 | 2.40 | 5.36 | 0.67 | 3.97 | 0.51 | 1.76 | 2.18 | 0.83 | 0.66 | 0.65 | 1.81 | 0.70 | 2.73 | 4.35 |
| OTU_90 | *Chrysocystis* sp. | 2.25 | 3.10 | 0.57 | 0.79 | 2.67 | 1.45 | 3.60 | 2.96 | 1.03 | 0.36 | 0.37 | 0.32 | 6.95 | 0.88 | 0.51 | 5.40 |
| OTU_177 | *Aureococcus anophagefferens* | 1.41 | 2.11 | 1.42 | 0.62 | 1.54 | 2.32 | 1.16 | 2.63 | 0.92 | 0.48 | 0.39 | 1.11 | 0.48 | 1.04 | 0.35 | 0.89 |
| OTU_3029 | *Aureococcus anophagefferens* | 2.40 | 0.75 | 2.61 | 1.58 | 0.73 | 2.71 | 1.85 | 3.47 | 0.85 | 1.29 | 0.26 | 0.85 | 3.60 | 0.27 | 3.09 | 1.32 |
| OTU_3 | *Strobilidiidae_X* sp. | 3.18 | 4.31 | 7.57 | 2.03 | 79.53 | 2.11 | 3.30 | 1.69 | 0.45 | 0.79 | 0.45 | 0.21 | 3.55 | 4.67 | 6.99 | 1.42 |
| OTU_21 | *Strombidium* sp. | 3.53 | 1.75 | 5.03 | 2.47 | 1.20 | 1.35 | 1.03 | 2.77 | 1.63 | 2.49 | 0.55 | 2.66 | 3.66 | 2.72 | 4.14 | 3.39 |
| OTU_29 | *Laboea strobila* | 1.95 | 1.22 | 0.36 | 0.04 | 0.32 | 1.46 | 0.06 | 2.01 | 11.08 | 1.54 | 0.77 | 1.07 | 1.54 | 3.69 | 14.48 | 0.56 |
| OTU_35 | *Tetmemena pustulata* | 2.48 | 1.82 | 0.77 | 0.27 | 1.93 | 0.35 | 0.61 | 2.60 | 3.63 | 1.12 | 0.18 | 0.09 | 3.68 | 4.25 | 1.04 | 0.87 |
| OTU_44 | *Strombidiidae_X* sp. | 1.60 | 3.56 | 3.33 | 0.31 | 4.25 | 0.48 | 1.43 | 0.91 | 0.76 | 1.25 | 0.36 | 0.46 | 1.83 | 2.86 | 4.04 | 0.90 |
| OTU_66 | *Choreotrichia_XX* sp. | 1.50 | 3.74 | 1.38 | 0.20 | 1.85 | 0.23 | 1.37 | 1.43 | 1.64 | 1.62 | 0.44 | 0.64 | 2.51 | 3.47 | 3.15 | 0.89 |
| OTU_69 | *Oligotrichia_XX* sp. | 2.07 | 2.51 | 2.34 | 1.44 | 1.81 | 0.96 | 1.58 | 1.32 | 1.30 | 1.40 | 1.06 | 1.21 | 1.66 | 2.67 | 2.90 | 3.09 |
| OTU_134 | *Choreotrichia-1_X* sp. | 3.17 | 3.24 | 2.99 | 2.81 | 1.37 | 1.38 | 1.22 | 2.21 | 2.39 | 0.57 | 0.26 | 0.94 | 1.72 | 2.65 | 9.65 | 10.48 |
| OTU_162 | *Strombidiidae_X* sp. | 1.65 | 1.13 | 0.48 | 0.07 | 0.69 | 0.07 | 0.92 | 1.83 | 5.40 | 3.09 | 0.39 | 0.90 | 2.45 | 2.90 | 2.32 | 0.90 |
| OTU_169 | *Strombidiidae_X* sp. | 1.63 | 1.97 | 0.33 | 0.41 | 1.70 | 0.34 | 0.77 | 1.04 | 2.25 | 2.43 | 2.57 | 4.58 | 2.43 | 2.38 | 2.93 | 2.78 |
| OTU_192 | *Strombidiidae_X* sp. | 8.49 | 4.63 | 4.63 | 1.54 | 0.57 | 1.54 | 0.53 | 1.21 | 1.30 | 1.67 | 0.34 | 0.53 | 3.47 | 1.90 | 4.32 | 0.62 |
| OTU_293 | *Choreotrichia_XX* sp. | 2.57 | 0.56 | 4.05 | 2.32 | 0.44 | 0.73 | 0.61 | 1.10 | 1.54 | 1.12 | 0.43 | 3.77 | 3.09 | 0.90 | 2.70 | 5.85 |
| OTU_321 | *Eutintinnus fraknoi* | 2.54 | 1.61 | 1.80 | 0.17 | 0.33 | 0.77 | 0.32 | 5.40 | 13.90 | 2.06 | 0.55 | 1.54 | 4.63 | 2.90 | 0.39 | 1.54 |
| OTU_440 | *Strombidiidae_X* sp. | 2.90 | 3.20 | 3.09 | 1.25 | 2.27 | 1.54 | 1.05 | 0.64 | 3.24 | 0.22 | 0.42 | 0.69 | 1.99 | 2.32 | 1.29 | 10.81 |
| OTU_453 | *Choreotrichia_XX* sp. | 3.09 | 6.18 | 2.01 | 1.54 | 1.71 | 3.09 | 0.77 | 1.67 | 1.50 | 0.56 | 0.22 | 1.03 | 7.26 | 3.16 | 2.93 | 4.44 |
| OTU_1083 | *Pseudotontonia simplicidens* | 6.45 | 2.63 | 2.32 | 1.52 | 1.25 | 1.52 | 1.27 | 3.30 | 2.21 | 2.86 | 0.24 | 2.39 | 5.02 | 2.69 | 3.35 | 1.69 |
| OTU_1716 | *Pseudotontonia simplicidens* | 4.08 | 2.54 | 5.58 | 1.14 | 3.35 | 1.72 | 2.10 | 3.49 | 2.26 | 1.08 | 0.15 | 0.54 | 4.34 | 7.62 | 1.32 | 2.83 |
| OTU_1824 | *Laboea* sp. | 4.95 | 2.46 | 7.09 | 0.81 | 0.63 | 0.98 | 0.07 | 1.76 | 14.29 | 0.92 | 1.54 | 5.69 | 2.57 | 2.39 | 0.36 | 0.02 |
| OTU_2043 | *Spirotontonia turbinata* | 5.66 | 1.07 | 7.76 | 1.21 | 2.62 | 1.04 | 0.44 | 1.06 | 3.55 | 1.29 | 0.23 | 0.90 | 3.63 | 1.65 | 10.13 | 0.70 |
| OTU_2129 | *Strombidiidae_X* sp. | 4.56 | 2.41 | 2.95 | 1.26 | 0.95 | 1.51 | 0.94 | 2.98 | 2.67 | 2.14 | 0.81 | 3.30 | 3.46 | 3.79 | 3.11 | 1.05 |
| OTU_2340 | *Strombidiidae_X* sp. | 2.32 | 0.39 | 2.70 | 0.77 | 0.60 | 0.99 | 0.26 | 0.46 | 2.63 | 6.47 | 1.95 | 4.25 | 5.40 | 0.51 | 0.39 | 0.10 |
| OTU_2651 | *Laboea* sp. | 1.91 | 0.97 | 4.93 | 2.32 | 1.91 | 0.45 | 1.17 | 0.90 | 1.06 | 1.19 | 0.89 | 1.09 | 1.69 | 1.91 | 4.19 | 1.35 |
| OTU_2826 | *Strombidiidae_X* sp. | 2.98 | 1.54 | 7.34 | 4.92 | 3.86 | 2.04 | 2.54 | 2.17 | 1.21 | 0.79 | 0.39 | 0.88 | 3.00 | 3.95 | 1.50 | 3.92 |
| OTU_3070 | *Strombidiidae_X* sp. | 1.82 | 5.23 | 2.32 | 0.77 | 6.18 | 1.54 | 2.18 | 1.54 | 1.52 | 1.96 | 0.05 | 0.16 | 1.87 | 6.29 | 2.17 | 1.25 |
| OTU_3272 | *Strombidiidae_X* sp. | 6.04 | 2.87 | 2.19 | 0.88 | 0.91 | 0.90 | 1.39 | 1.00 | 2.70 | 1.93 | 0.07 | 4.01 | 5.79 | 2.32 | 1.93 | 2.08 |
| OTU_3956 | *Choreotrichia_XX* sp. | 6.02 | 5.83 | 0.88 | 0.90 | 1.01 | 0.58 | 0.58 | 1.54 | 1.35 | 1.77 | 0.06 | 3.58 | 3.20 | 4.05 | 1.74 | 3.44 |
| OTU_4053 | *Strombidium* sp. | 5.11 | 2.49 | 2.73 | 0.70 | 0.65 | 1.05 | 0.58 | 1.87 | 1.50 | 4.27 | 0.43 | 2.84 | 3.63 | 4.04 | 4.69 | 1.32 |

# Supplementary Figures


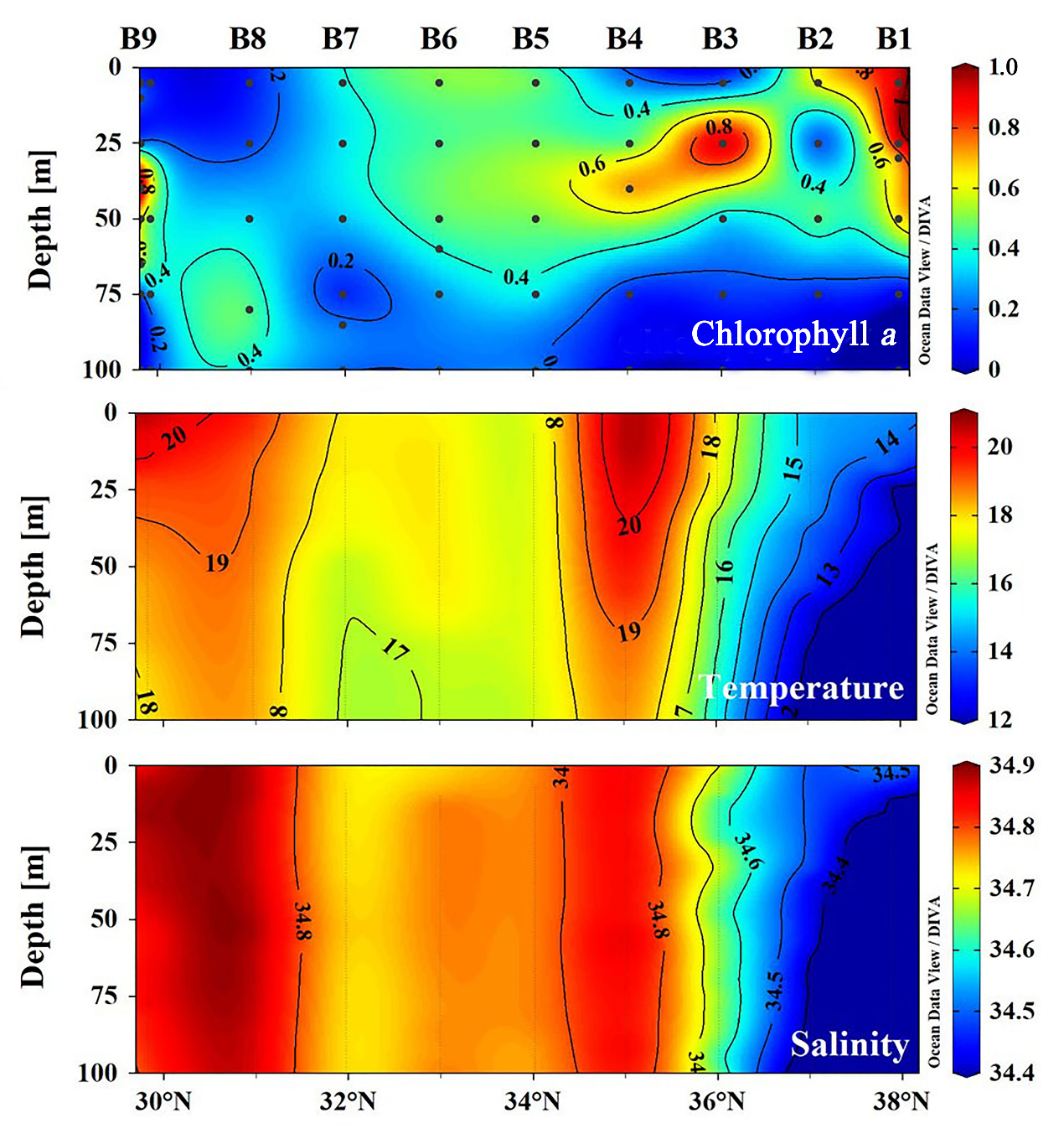


**Figure S1.** Depth distribution of chlorophyll *a* (Chl *a*, μg L^-1^) and vertical profiles of temperature (°C) and salinity upper 100 m across sampling stations from the northernmost B1 to B9 in the northwestern Pacific Ocean. Chl *a* concentrations were derived from the HPLC-based pigment analysis. Black dots represent the sampling points.


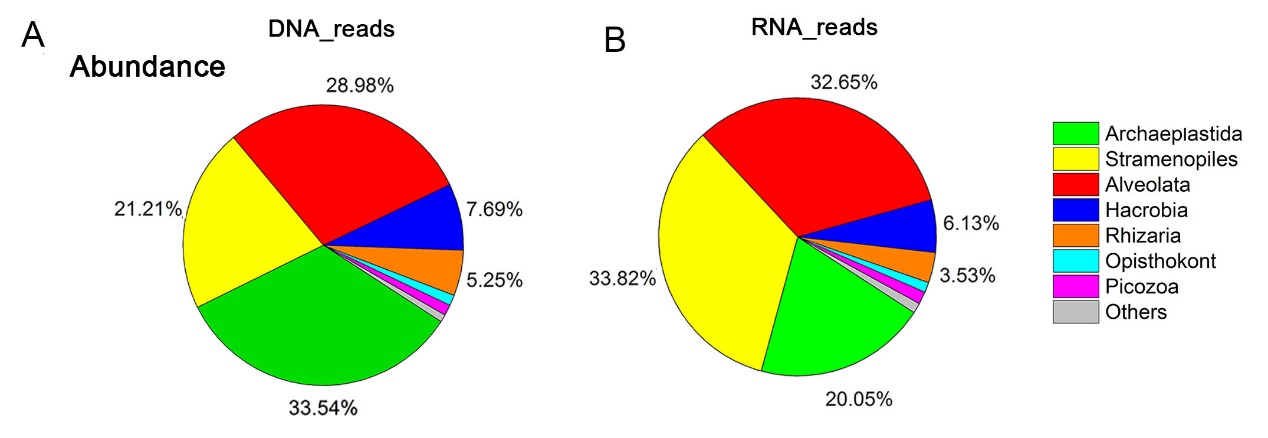


**Figure S2.** Relative abundance of picoeukaryotes at super-group level for 16 samples based on the DNA (A) and RNA (B) surveys. Others refer to the remaining super-groups with low relative abundance, including Excavata, Amoebozoa and Apusozoa.


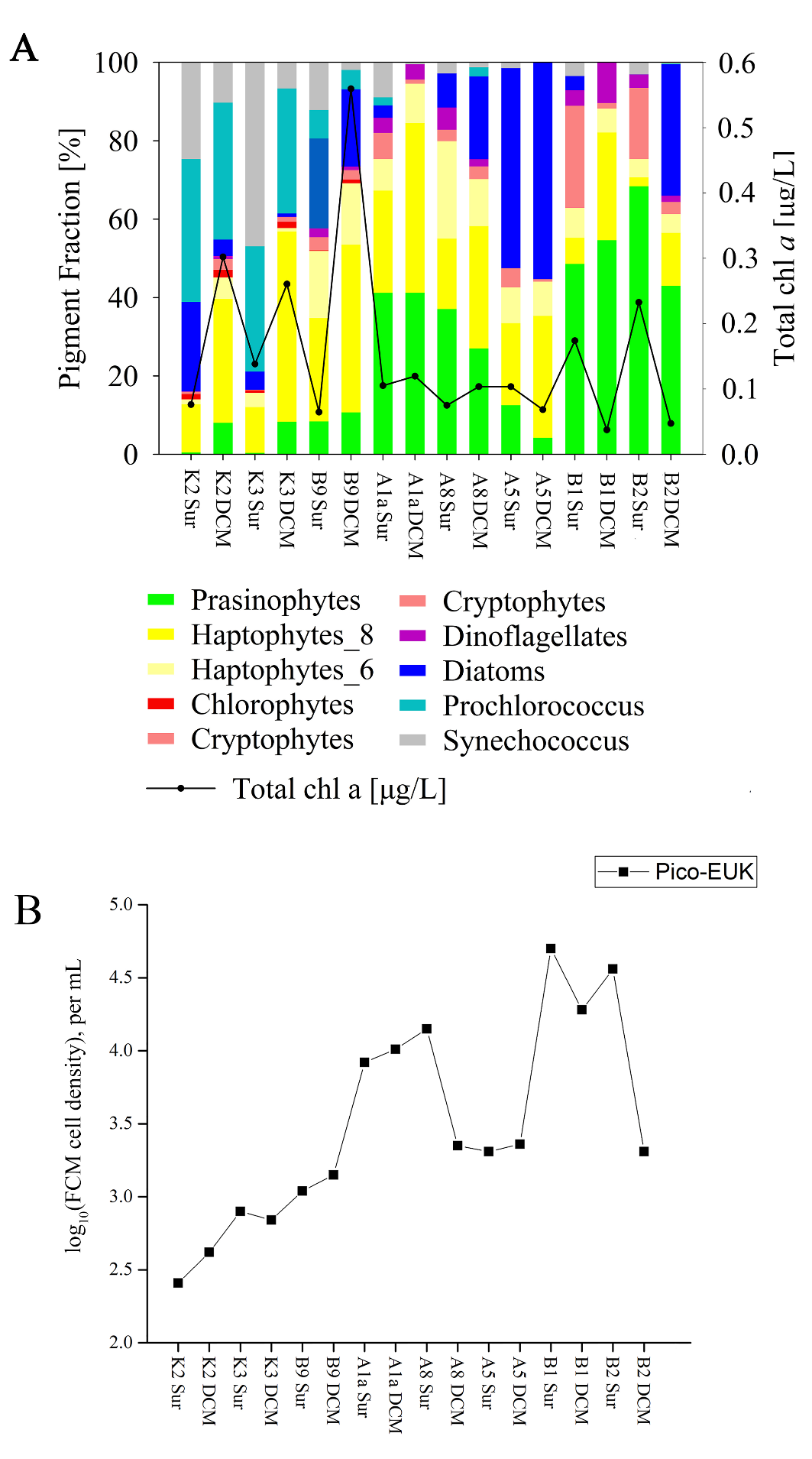


**Figure S3.** Relative contributions of phytoplankton derived from pigment data using CHEMTAX analysis. Point line indicated the total pigment concentration at each sample (A). Cell abundance of picoeukaryotes in each sample enumerated using a Becton Dickinson FACSCalibur cytometer (B).


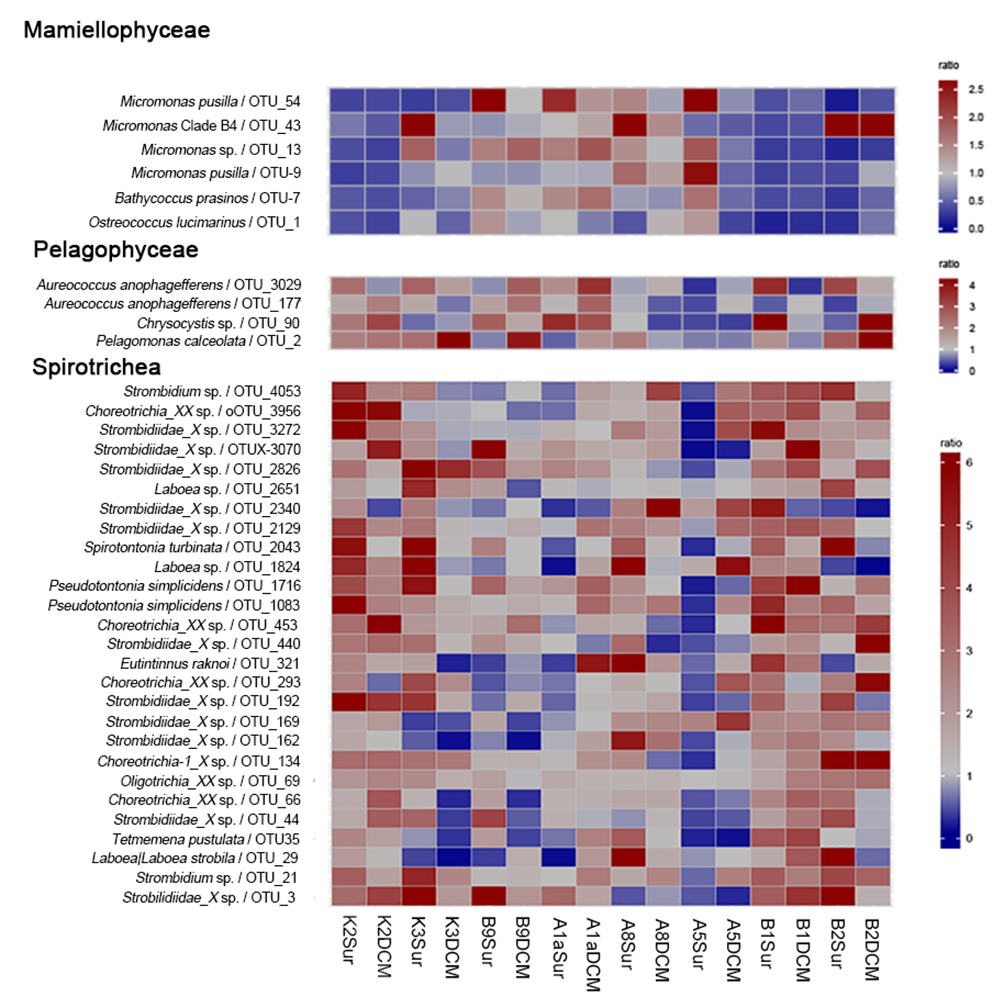


**Figure S4.** Heatmap of the relative metabolic activity (i.e., RNA: DNA ratios) of Mamiellophyceae, Pelagophyceae and Spirotrichea species among samples. The relative metabolic activity is indicated according to the scale bar. The dark red indicates high relative metabolic activity, and the dark blue indicates low relative metabolic activity.
